# First person – Rebecca Rolfe

**DOI:** 10.1242/dmm.049040

**Published:** 2021-04-22

## Abstract

First Person is a series of interviews with the first authors of a selection of papers published in Disease Models & Mechanisms, helping early-career researchers promote themselves alongside their papers. Rebecca Rolfe is first author on ‘Joint development recovery on resumption of embryonic movement following paralysis’, published in DMM. Rebecca is a Research and Teaching Fellow in the lab of Prof. Paula Murphy at Trinity College Dublin, Ireland, investigating the role environmental cues play in the correct development of cells and tissues during embryonic development.


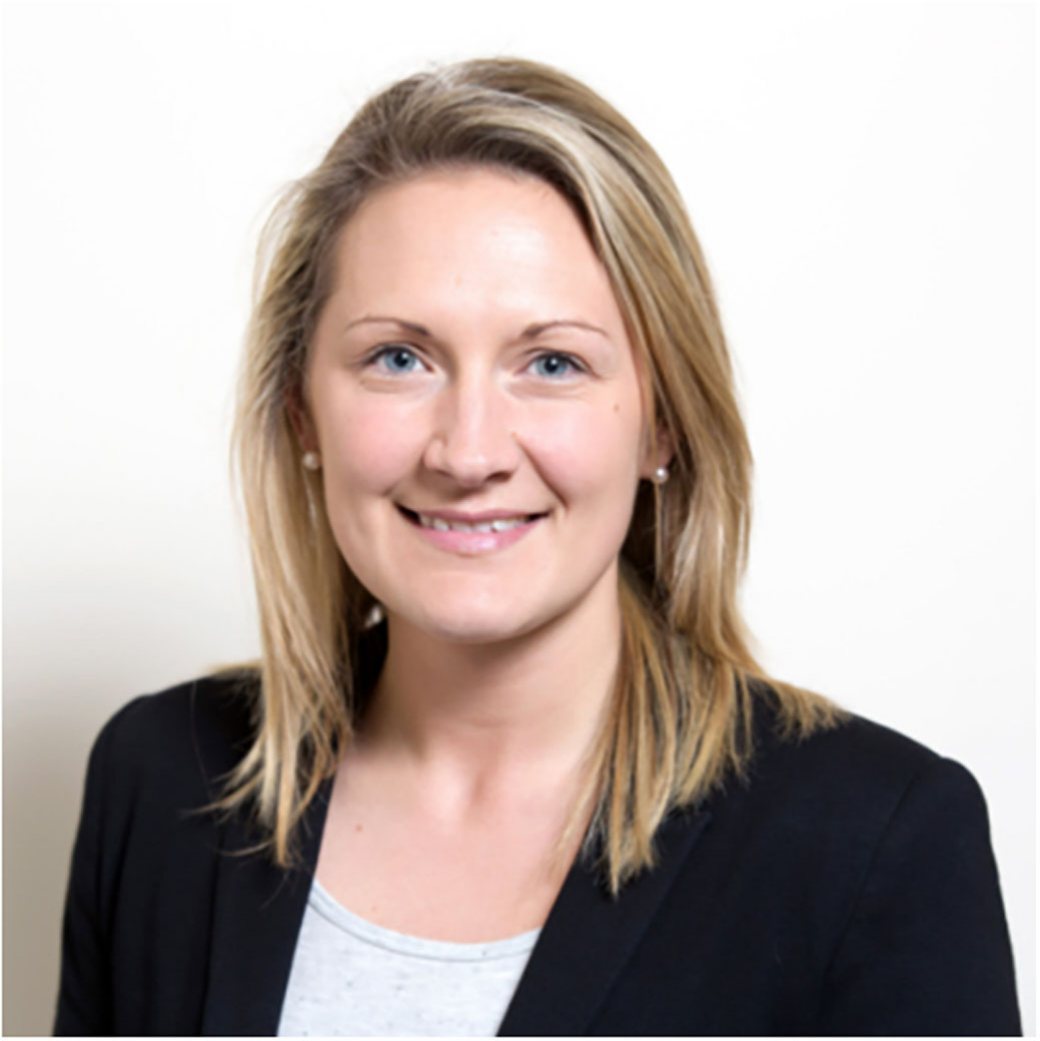


**Rebecca Rolfe**

**How would you explain the main findings of your paper to non-scientific family and friends?**

Foetal movement in the uterus is a normal part of a healthy pregnancy but reduced or absent foetal movement can lead to problems with the development of bones and joints, including joint dysplasia and temporary brittle bone disease in infants. Chicken embryos provide a good model system to help us understand how this occurs, as they develop within an external egg, which can be viewed and manipulated experimentally. The skeletal system is very complex and although we know some of the cellular and molecular processes that are involved when embryo movement is restricted, we know very little about the capacity for the system to recover following periods of immobility during development. In this study, we used developing chicken embryos to investigate whether movement resumes after a defined period of movement restriction early in development, which has relevance to possible therapeutic interventions for human conditions – can joint and spine formation recover? We compared resumption of normal movement to hyperactive movement and showed that embryonic movement following a period of immobility can partially recover aspects of joint development, and limited recovery was observed in the spine. We also found that hyperactive movement led to greater improvement in joint development, especially at the hip, indicating that clinical conditions resulting from reduced activity of the foetus in the womb could be ameliorated with physical movement or manipulation even after an initial problem becomes apparent. Outcomes from this work could inform therapeutic approaches to ameliorate the effects of human reduced foetal movement *in utero*.

**What are the potential implications of these results for your field of research?**

This work demonstrates that movement post paralysis can partially recover specific aspects of joint development, which could inform therapeutic approaches to ameliorate the effects of human foetal immobility *in utero*. It is the first study to integrate analysis of the appendicular and axial skeleton, providing insight into the differential plasticity of the skeletal system and the potential for recovery. The work presented in this study provides a detailed morphological description of the response within the skeletal system to restoration of movement following a period of immobility. In particular, it shows that multiple aspects of joint development, disturbed when mechanical stimulation is removed, can recover when movement resumes. Further examination of the molecular and cellular mechanisms of this partial recovery will be important to further our understanding of the mechanisms involved in the interplay of mechanical information with cellular differentiation. Information from this research could inform clinical assessment of congenital conditions in which short periods of paralysis occur *in utero*.

“[…] movement post paralysis can partially recover specific aspects of joint development, which could inform therapeutic approaches to ameliorate the effects of human foetal immobility *in utero*.”

**What are the main advantages and drawbacks of the model system you have used as it relates to the disease you are investigating?**

The main advantage of the chick embryonic model used in this study is the amenability to disturb the mechanical environment during specific periods of development. This benefit was taken advantage of in this study to both alter normal movement, inducing paralysis, and to induce hyperactive movement. This system models reduced foetal movement *in utero*, resulting in skeletal abnormalities in humans. Directly investigating reduced foetal movement *in utero* is challenging, and the use of experimental animal models have helped to inform the impact of reduced movement on skeletogenesis, and have established that mechanical forces produced by embryonic movements are crucial for normal skeletal development.

**What has surprised you the most while conducting your research?**

The recovery in aspects of particular joints in the skeleton during embryonic development following a period of immobility was a surprise during this research and highlights the potential plasticity in the response of certain cells and tissues during their patterning to environmental cues. This is of great interest as it could have implications for human *in utero* development in which periods of immobility arise in the foetus during normal development.

**Describe what you think is the most significant challenge impacting your research at this time and how will this be addressed over the next 10 years?**

Although the use of animal models to study conditions of human development is of immense value, in our context both due to the amenability to external manipulation and the recent developments in the field of genetics, the translation to clinical application will still take time. Our study highlights that continued understanding of *in utero* conditions requires a more integrated approach with clinical researchers to bridge the gap between basic and applied research. I believe increasing networks of scientists and clinicians are required to expand communication of both the basic research and the clinical need to further our understanding of congenital conditions into the future.

**Figure:**
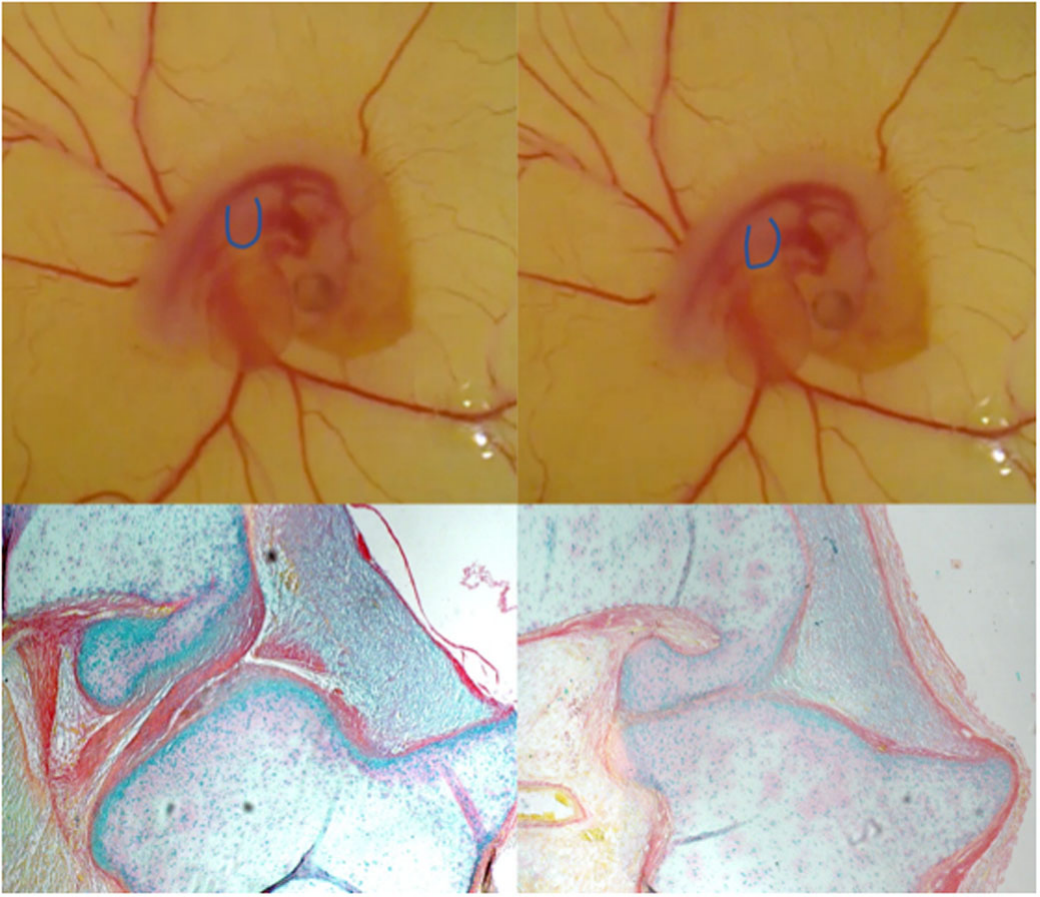
**Snapshots of a chick embryo developing in *ex ovo* culture condition (top row), showing how the onset of embryo movement and limb displacement (illustrated with outlines overlaid on the developing forelimb) was monitored over time in this study (top left, at the onset of movement, and top right, after movement). The bottom row shows histology sections of knee joint with normal embryo movement (bottom left) and absence of embryonic movement (bottom right).**

**What changes do you think could improve the professional lives of early-career scientists?**

I would love to see the standard implementation of longer-term research contracts with associated support within universities, like childcare and family support, health insurance and long-term career planning support to coincide with postdoctoral contracts. Postdoctoral research or research fellow positions are traditionally seen as a transition period between postgraduate research and faculty positions. As competition for permanent faculty positions is growing, and the availability of these positions is less, there becomes a time when a major determining factor for an individual to stay in academia is whether they can both psychologically and socioeconomically afford to sustain the career path. More opportunities for early career scientists, with increased fellowships and early career awards that promote independent research goals, are required. The acknowledgement that a majority of female postdocs are of child-bearing age, and of the pressures and responsibilities that brings, can interfere greatly with the generation of research output required for this career stage and thus impact career progression. Being able to think independently and ask questions in order to enhance knowledge of a topic is central for researchers, and it should not be stifled by external factors limiting their success.

“Being able to think independently and ask questions in order to enhance knowledge of a topic is central for researchers, and it should not be stifled by external factors limiting their success.”

**What's next for you?**

In an experimental setting, we are also looking at the patterning of embryonic cells and the implication of mechanics on other aspects of musculoskeletal development. I will continue my research on mechanobiological of embryonic development as I apply for independent funding and continue to search for academic positions. In parallel, I will continue to provide support to my growing family and hope to motivate my daughter to continually ask questions and help her to discover the world around us.
